# Surgical Versus Conservative Treatment for Intra‐Articular Distal Radius Fractures With Loss of Reduction in Elderly Patients

**DOI:** 10.1111/os.70338

**Published:** 2026-05-25

**Authors:** Bekir Karagoz, Hünkar Cagdas Bayrak, Durmus Ekin Dincer

**Affiliations:** ^1^ Eskisehir City Hospital Department of Orthopedics and Traumatology Eskisehir Turkey; ^2^ Cekirge State Hospital Department of Orthopedics and Traumatology Bursa Turkey; ^3^ Gaziantep City Hospital Department of Orthopedics and Traumatology Gaziantep Turkey

**Keywords:** conservative treatment, distal radius fracture, elderly patients, loss of reduction, surgical fixation, volar plate

## Abstract

**Objective:**

The optimal treatment strategy for intra‐articular distal radius fractures with loss of reduction in elderly patients remains unclear, particularly in AO/OTA 23‐C1 and C2 fracture types. This study aimed to compare the clinical and radiological outcomes of surgical versus conservative treatment in elderly patients who developed secondary displacement after initial reduction.

**Methods:**

In this retrospective multicenter study, 127 patients aged ≥ 65 years with AO/OTA 23‐C1/C2 distal radius fractures and secondary displacement during follow‐up were analyzed. Patients were managed either with continued conservative treatment (*n* = 78) or delayed surgical intervention (*n* = 49). Radiological parameters (volar tilt, radial inclination, ulnar variance, and articular step‐off) and functional outcomes were evaluated at 3 and 12 months using the Quick Disabilities of the Arm, Shoulder, and Hand (QuickDASH) score and the Patient‐Rated Wrist Evaluation (PRWE). Grip strength was assessed bilaterally at the 12‐month follow‐up. Independent predictors of conversion to surgical treatment were identified using multivariate logistic regression analysis.

**Results:**

At the 3‐month follow‐up, the surgical group demonstrated significantly better radiological alignment and lower QuickDASH and PRWE scores compared with the conservative group (*p* < 0.01). Grip strength on the injured side was similar between groups; however, relative strength loss compared with the uninjured side was significantly lower in the surgical group (*p* = 0.001). At 12 months, no significant differences were observed between groups in either radiological or functional outcomes. Implant‐related irritation was more frequent in the surgical group (*p* = 0.01), whereas malunion occurred more commonly in the conservative group (*p* = 0.047). Independent predictors of surgical intervention included AO/OTA 23‐C2 fracture type (OR: 2.677; *p* = 0.023) and progressive deterioration in alignment during follow‐up (OR: 15.1; *p* = 0.001).

**Conclusion:**

Surgical treatment provided superior short‐term radiological and functional outcomes in elderly patients with distal radius fractures complicated by loss of reduction; however, long‐term outcomes were comparable between treatment strategies. Treatment decisions should be individualized, taking into account fracture pattern, alignment stability during follow‐up, and patient‐specific factors.

## Graphical abstract

Population: Elderly patients (≥ 65 years) with AO/OTA 23‐C1/C2 distal radius fractures and secondary loss of reduction. Comparison: Conservative treatment vs. surgical fixation. Findings: Surgery provides superior short‐term radiological and functional outcomes; long‐term results are comparable. Key Predictors: Fracture pattern (C2) and reduction quality.

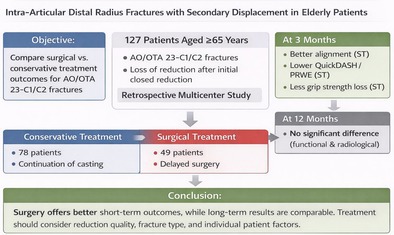

## 1 Introduction

Distal radius fractures are among the most common upper extremity injuries in the elderly population due to the combined effects of osteoporosis and low‐energy trauma such as falls [1, 2]. These fractures can lead to functional limitations, reduced quality of life, and increased dependency. Treatment decisions are based on factors such as the patient's age, activity level, comorbidities, and fracture morphology, which guide the selection between conservative and surgical approaches [3].

Conservative treatment typically involves closed reduction and immobilization with a cast. However, maintaining reduction during follow‐up can be difficult, especially in patients who initially receive non‐operative management [4, 5]. In such cases, deciding whether to proceed with surgical intervention remains controversial. This is particularly relevant for intra‐articular fractures that are not highly comminuted, such as *Arbeitsgemeinschaft für Osteosynthesefragen/Orthopedic Trauma Association* (AO/OTA) type 23‐C1 and C2 fractures, where there is no consensus on the optimal treatment strategy [6, 7].

Several studies have suggested that surgical fixation leads to better early radiological outcomes in distal radius fractures [8, 9]. However, the long‐term impact of this radiological improvement on functional recovery is still uncertain. The timing and necessity of surgical conversion after loss of reduction are also debated. While some retrospective studies indicate that conservative treatment may provide satisfactory functional results in elderly patients despite displacement [4, 10], others report that surgical intervention offers superior early radiographic alignment in intra‐articular fractures [11, 12, 13]. Importantly, most available studies have primarily focused on comparing initial treatment strategies at the time of injury. In contrast, a frequent and clinically challenging scenario in daily practice involves elderly patients who initially undergo closed reduction and cast immobilization but subsequently develop secondary displacement during follow‐up. For this specific subgroup of AO/OTA type 23‐C1 and C2 fractures, the optimal management strategy and the factors that should prompt conversion to surgical treatment remain insufficiently defined. Identifying clinically relevant predictors such as fracture pattern and reduction quality is therefore essential to better inform decision‐making in this setting.

The aims of this study were: (i) to compare radiological and functional outcomes of conservative and surgical treatment in patients aged ≥ 65 years with AO/OTA 23‐C1/C2 distal radius fractures who developed loss of reduction after initial reduction; (ii) to assess temporal differences between early and late outcomes; and (iii) to identify independent predictors associated with conversion to surgical treatment. Our hypothesis was that fracture pattern and reduction quality influence the need for surgical intervention and affect the functional and radiological outcomes.

## 2 Materials and Methods

### 2.1 Study Design and Patient Selection

This study was designed as a retrospective, multicenter, observational cohort study conducted at three orthopedic and traumatology departments. The study protocol was approved by the ethics committee of the coordinating center, and the study was carried out in accordance with the principles of the Declaration of Helsinki.

Between January 2018 and December 2023, a total of 377 patients aged 65 years or older who were diagnosed with AO/OTA type 23‐C1 or 23‐C2 distal radius fractures and treated at one of the three participating centers were initially screened.

As an inclusion prerequisite, all patients underwent closed reduction followed by below‐elbow cast immobilization and achieved an acceptable initial reduction based on predefined radiographic criteria. Inclusion criteria were defined as follows: (i) patients aged ≥ 65 years with AO/OTA type 23‐C1 or 23‐C2 distal radius fractures; (ii) initial management with closed reduction and cast immobilization; (iii) achievement of acceptable initial reduction; and (iv) development of loss of reduction during follow‐up. Patients who did not achieve acceptable initial reduction were not eligible for inclusion.

**FIGURE 1:**
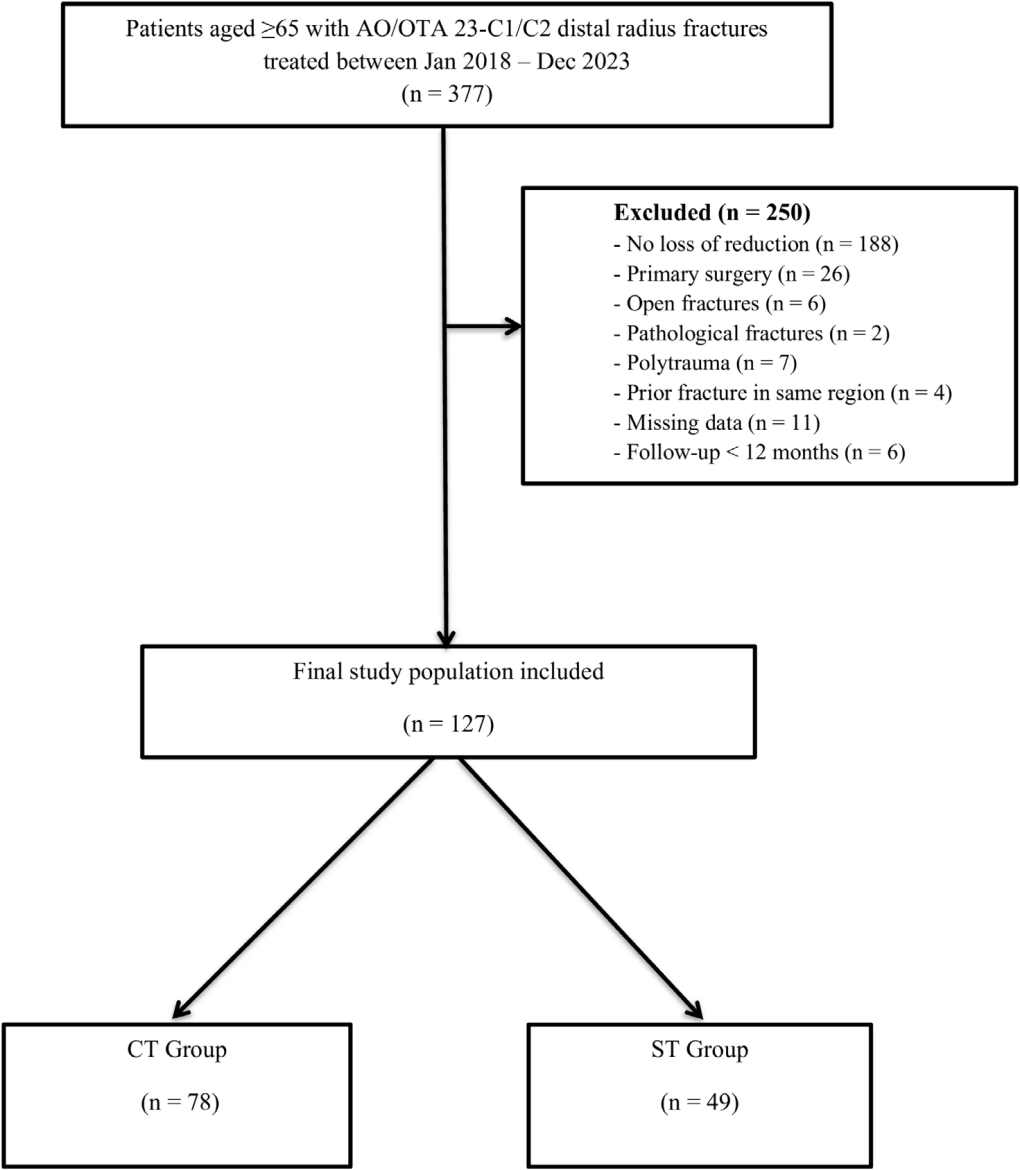
Flowchart of patient selection.

Exclusion criteria were defined as follows: (i) absence of loss of reduction during follow‐up; (ii) primary surgical treatment at initial presentation; (iii) open or pathological fractures; (iv) polytrauma; (v) previous fracture in the same region; (vi) missing clinical or radiological data; and (vii) follow‐up duration of less than 12 months. Patients who developed loss of reduction during follow‐up were subsequently divided into two groups according to the treatment strategy: continued conservative treatment (CT group) and delayed surgical treatment (ST group). The patient selection process, inclusion and exclusion criteria, and group allocation algorithm are summarized in Figure 1.

Loss of reduction was defined as secondary displacement observed during follow‐up, based on the presence of one or more of the following radiographic criteria: > 10° dorsal angulation in volar tilt, > 2 mm increase in ulnar variance, > 5° decrease in radial inclination, or > 1 mm articular step‐off [5]. These radiographic parameters were used to identify loss of reduction but did not constitute absolute indications for surgical intervention.

### 2.2 Treatment Management

All patients were evaluated by an orthopedic specialist upon admission to the emergency department, and closed reduction was performed as the initial intervention. Following successful reduction, all cases were immobilized with a below‐elbow cast, which served as the standard initial treatment protocol. Patients were monitored through scheduled outpatient visits: weekly during the first month after reduction, monthly until the third month, and again at the sixth and twelfth months. At each follow‐up, posteroanterior and lateral wrist radiographs were obtained to assess maintenance of reduction. In patients who developed loss of reduction, the decision to proceed with surgery was made based on a combination of clinical and radiological criteria. The surgical decision was based on a multidisciplinary assessment, considering the patient's symptoms, physical examination findings, and fracture morphology. These criteria influencing surgical intervention were thoroughly discussed with the patient during the treatment planning process, and the final treatment strategy was implemented after obtaining informed consent.

**FIGURE 2:**
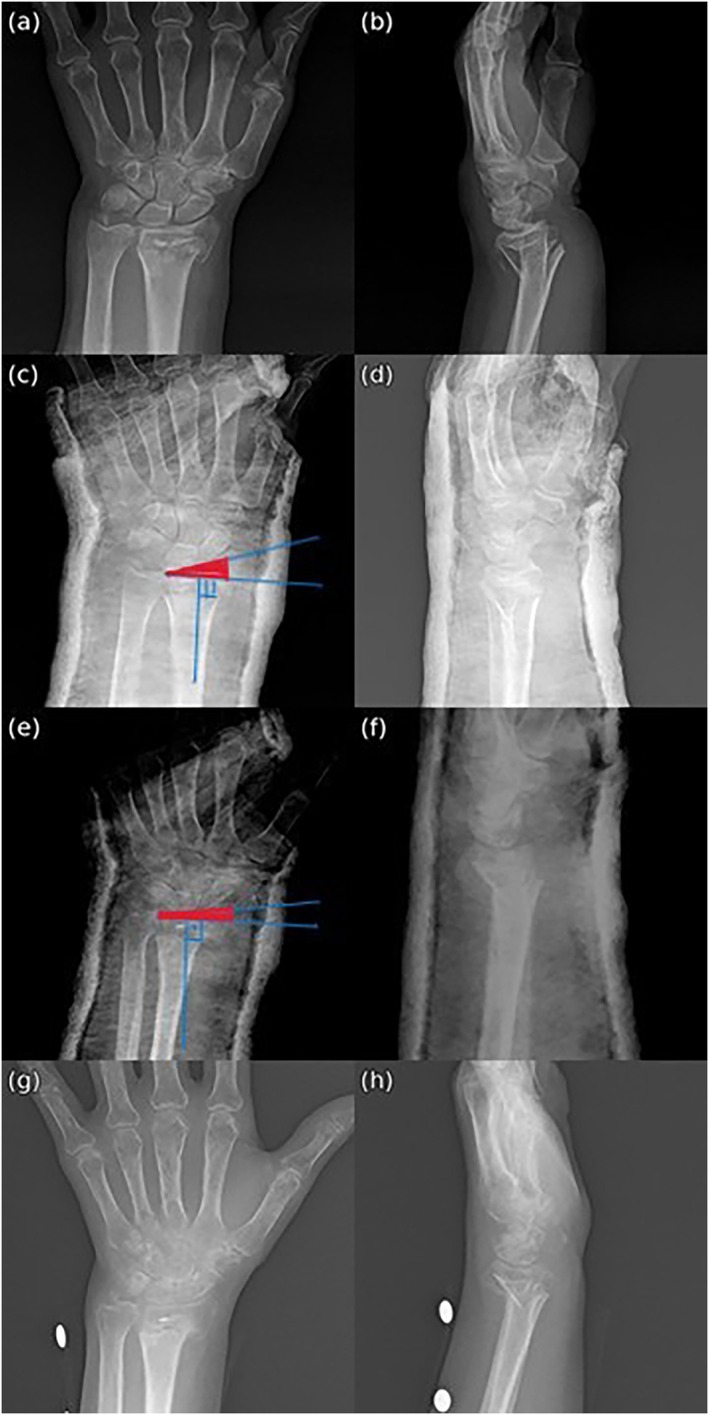
Radiographic series of a 78‐year‐old female patient with an intra‐articular distal radius fracture (AO/OTA type 23‐C2) who developed secondary loss of reduction during follow‐up. (a, b) At initial presentation, anteroposterior and lateral radiographs demonstrate the fracture pattern. (c, d) Immediately after closed reduction, radiographs obtained in a below‐elbow cast show acceptable alignment. (e, f) At 2‐week follow‐up, radiographs demonstrate secondary loss of reduction. Despite radiographic displacement, surgical conversion was not performed due to the patient's high anesthetic risk and comorbidities. (g, h) At final follow‐up (12 months), radiographs demonstrate fracture union with malunion. Radial inclination is demonstrated on selected images (c, e), showing a decrease over time.

All patients in the CT group were managed using a standardized non‐operative protocol (Figure 2). They were immobilized with a below‐elbow cast for approximately 5–6 weeks, applied in a neutral wrist position. The cast allowed preservation of motion in the remaining upper extremity joints. At each outpatient visit, fracture healing and the stability of the reduction were evaluated through radiographic imaging and physical examination. No patients in the conservative treatment group underwent re‐reduction during follow‐up.

**FIGURE 3:**
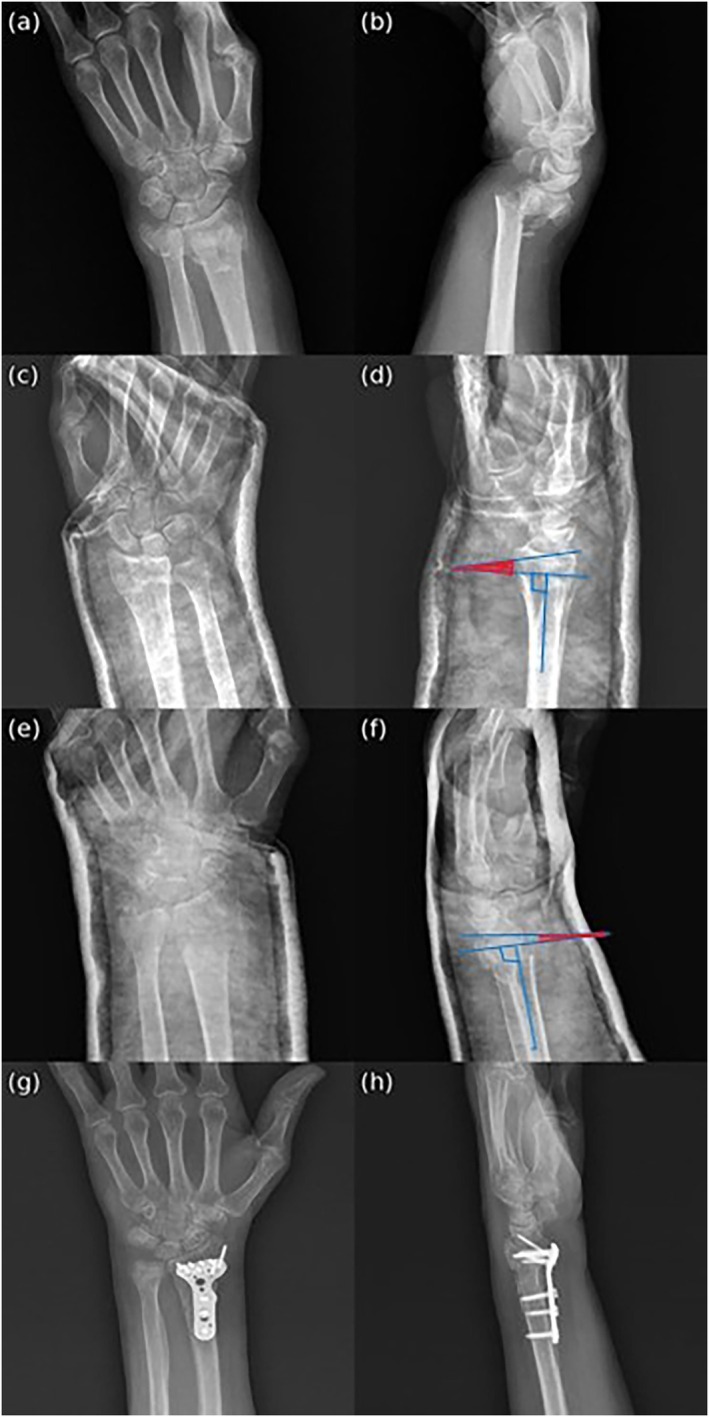
Radiographic series of a 74‐year‐old male patient with an intra‐articular distal radius fracture (AO/OTA type 23‐C2) who developed secondary loss of reduction and was treated surgically. (a, b) At initial presentation, radiographs demonstrate the fracture pattern. (c, d) Immediately after closed reduction, radiographs show temporary acceptable alignment. (e, f) At 2‐week follow‐up, radiographs demonstrate progressive loss of reduction. (g, h) At final follow‐up (12 months), postoperative radiographs demonstrate fracture fixation with a volar locking plate and restoration of alignment. Volar tilt is illustrated on selected images (d, f), demonstrating deterioration prior to surgical intervention.

In the ST group, all procedures were performed by orthopedic surgeons with at least 5 years of experience in orthopedic trauma surgery, under appropriate anesthesia. The choice of surgical technique was individualized based on fracture characteristics, bone quality, soft tissue condition, and patient‐related factors. Three different surgical techniques were utilized. First, volar plate fixation involved open reduction via the Henry approach and internal fixation with anatomically contoured locking plates; screw positions were confirmed intraoperatively by fluoroscopy (Figure 3). Second, Kirschner wire (K‐wire) fixation was performed by percutaneously inserting two or three cross or parallel wires under fluoroscopic guidance following closed reduction; K‐wires were removed at postoperative week 6. Third, external fixation was applied using a dorsal bridging technique between the radial diaphysis and the second metacarpal, and in some cases was supplemented with additional K‐wires. Following surgery, early finger mobilization was encouraged in all patients. Standard wound care was provided, and sutures were typically removed between days 12 and 14 postoperatively.

Regardless of treatment group, once the immobilization period was completed, all patients initiated the same rehabilitation program to support functional recovery. This program consisted of active range‐of‐motion exercises targeting wrist flexion‐extension, radial and ulnar deviation, and pronation‐supination; isometric grip‐strengthening exercises; and functional training aimed at improving performance in daily activities. In the CT group, exercises began immediately after cast removal. In the ST group, exercises were generally initiated around the fourth postoperative week, depending on the surgical method and fracture stability. During follow‐up visits, patients received personalized home exercise instructions, and referral to physical therapy units was made when necessary.

### 2.3 Clinical Evaluation

Demographic, radiological, and clinical follow‐up data of all included patients were obtained from the digital medical record systems of the participating hospitals. Trauma mechanism was categorized as low‐energy or high‐energy according to the injury context. Low‐energy trauma was defined as falls from standing height or lower, whereas high‐energy trauma included falls from height, traffic accidents, or other high‐impact mechanisms. Radiological evaluation was based on standard posteroanterior and lateral wrist radiographs obtained during follow‐up visits. Four key parameters were measured: volar tilt, radial inclination, ulnar variance, and articular step‐off [14, 15, 16]. Reduction quality was classified as anatomic or inadequate based on post‐reduction radiographic parameters. Anatomic reduction was defined as restoration of volar tilt, radial inclination, ulnar variance, and articular congruity within accepted alignment limits. Reduction was considered inadequate when one or more of these parameters exceeded accepted thresholds following initial reduction. Malunion was defined as fracture union with persistent dorsal angulation and/or other radiographic parameters exceeding accepted alignment limits at final follow‐up. Fractures were classified according to the AO/OTA system. All classifications and measurements were performed independently by two orthopedic surgeons, blinded to each other's assessments, using the Picture Archiving and Communication System (PACS). In cases of disagreement regarding fracture classification, a third orthopedic specialist reviewed the radiographs and made the final decision. To assess interobserver reliability, intraclass correlation coefficient (ICC) analysis was performed. ICC values for volar tilt, radial inclination, ulnar variance, and articular step‐off were found to be 0.89, 0.91, 0.87, and 0.88, respectively, indicating good to excellent agreement across all parameters. Functional outcomes were assessed using the Quick Disabilities of the Arm, Shoulder and Hand (QuickDASH) and the Patient‐Rated Wrist Evaluation (PRWE) scoring systems. Both instruments are standardized, patient‐reported outcome measures specifically designed to evaluate upper extremity and wrist‐related disability. Scores for both QuickDASH and PRWE range from 0 to 100, with higher scores indicating greater disability, increased pain, and poorer functional outcomes [17, 18]. These scores were recorded during outpatient visits at the 3rd and 12th months. In addition, grip strength was measured bilaterally on both the injured and uninjured sides at the 12‐month follow‐up. Grip strength was assessed using a Jamar dynamometer with patients in a seated position and the elbow flexed to 90 degrees; the mean of three consecutive measurements was recorded [19]. Wrist range of motion, including flexion, extension, pronation, and supination, was assessed at the 3rd and 12th months during outpatient visits using a standard goniometer. Throughout the treatment and follow‐up period, all complications were systematically monitored and documented.

### 2.4 Statistical Analysis

All statistical analyses were performed using IBM SPSS Statistics version 25.0 (IBM Corp., Armonk, NY, USA). Descriptive statistics—including mean, standard deviation, minimum–maximum values, frequency, and percentage—were used to summarize the characteristics of the data. The Kolmogorov–Smirnov test was employed to assess the distribution of continuous variables. For normally distributed data, comparisons between groups were made using the independent samples *t*‐test. For non‐normally distributed data, the Mann–Whitney *U* test was applied. Categorical variables were compared using the Chi‐square test. In addition, a multivariate logistic regression analysis was performed to identify independent predictors influencing the decision to proceed with surgical treatment. A *p* < 0.05 was considered statistically significant in all analyses.

## 3 Results

### 3.1 Patient Characteristics

After applying the predefined inclusion and exclusion criteria, 127 patients were included in the final analysis. Of these patients, 78 were assigned to the CT group, while 49 were assigned to the ST group following the development of loss of reduction. The mean age of the patients included in the study was 76.9 ± 7.4 years. Of the total cohort, 72 patients (57%) were female, and 55 (43%) were male. The mean follow‐up duration was 14.2 ± 2.8 months. In patients who converted to surgical treatment, the mean time from initial reduction to surgical intervention was 2.27 ± 0.97 weeks, with a minimum of 1 week and a maximum of 4 weeks.

Demographic characteristics, fracture types, comorbid systemic conditions, and American Society of Anesthesiologists (ASA) scores of the patients are presented in Table 1. There were no statistically significant differences between the surgical and conservative treatment groups in terms of age, sex, dominant side, mechanism of injury, AO/OTA classification, comorbidities, or ASA scores.

**TABLE 1:** Baseline characteristics of the study population.

Variable	CT group (*n* = 78)	ST group (*n* = 49)	*p*
Age (years), mean ± SD	77.3 ± 7.1	76.8 ± 7.3	0.710
Gender, *n* (%)			0,935
Male	34 (43.6%)	21 (42.9%)	
Female	44 (56.4%)	28 (57.1%)	
Dominant hand injured, *n* (%)	34 (43.6%)	24 (49%)	0,681
Mechanism of injury, *n* (%)			0.466
Low energy	70 (89.7%)	41 (83.7%)	
High energy	8 (10.3%)	8 (16.3%)	
Fracture type (AO/OTA), *n* (%)			0.052
23‐C1	50 (64.1%)	22 (44.9%)	
23‐C2	28 (35.9%)	27 (55.1%)	
Comorbidities, *n*			
Hypertension	51	27	0.331
Diabetes Mellitus	34	20	0.902
Parkinson's disease	4	3	0.811
Alzheimer's disease	8	5	0.995
Renal failure	6	3	0.737
Osteoporosis	23	10	0.256
Chronic obstructive pulmonary disease	5	2	0.576
Coronary heart disease	13	7	0.914
ASA score, *n* (%)			0.106
1	2 (2.6%)	4 (8.2%)	
2	49 (62.8%)	24 (49.0%)	
3	24 (30.8%)	21 (42.9%)	
4	3 (3.8%)	0 (0.0%)	

*Note:* Continuous variables were compared using the independent samples *t*‐test, and categorical variables were analyzed using the Pearson Chi‐square test (χ^2^ values are reported).

### 3.2 Radiological and Functional Outcomes

As summarized in Table 2, the ST group demonstrated significantly better radiological parameters at the 3‐month follow‐up, including volar tilt, radial inclination, and ulnar variance (all *p* < 0.001). Similarly, functional scores were significantly better in the ST group at 3 months, with lower QuickDASH and PRWE scores compared to the CT group (*p* = 0.006 and *p* = 0.003, respectively) (Table 3). However, these differences were no longer statistically significant at the 12‐month evaluation. Range of motion in the wrist joint showed comparable results between the two groups at both 3 and 12 months (Table 3). Regarding grip strength, absolute values measured on the injured side at 12 months were comparable between the two groups. However, when grip strength loss was evaluated relative to the contralateral (non‐injured) side, the conservative treatment group demonstrated a significantly greater deficit compared to the surgical treatment group (3.43 ± 0.87 kg vs. 2.81 ± 0.83 kg, *p* = 0.001) (Table 3). When expressed as a percentage of the contralateral side, this corresponded to an average grip strength loss of 13.7% in the conservative treatment group versus 11.4% in the surgical treatment group, indicating a relative preservation of grip strength following surgical treatment.

**TABLE 2:** Comparison of radiological parameters between conservative and surgical groups.

Variable	CT group (*n* = 78)	ST group (*n* = 49)		*p*
Volar tilt (°)				
After reduction	11.37 ± 3.08	10.92 ± 2.83		0.394
At 3 months	7.36 ± 3.37	9.37 ± 2.98		0.001^a^
Radial inclination (°)				
After reduction	22.62 ± 3.37	21.96 ± 3.17		0.108
At 3 months	18.46 ± 3.34	20.39 ± 3.01		0.001^a^
Ulnar variance (mm)				
After reduction	−0.42 ± 0.94	−0.26 ± 0.86		0.213
At 3 months	2.81 ± 1.53	0.98 ± 1.20		0.001^a^
Articular step‐off (mm)				
After reduction	0.41 ± 0.50	0.39 ± 0.49		0.802
At 3 months	1.10 ± 0.50	0.84 ± 0.47		0.004^a^

^a^ *p* < 0.05 was considered statistically significant. Continuous variables were compared using the independent samples *t*‐test or the Mann–Whitney *U* test, as appropriate (*t* values are reported).

**TABLE 3:** Comparison of functional and clinical outcomes between conservative and surgical groups.

Variable	CT group (*n* = 78)	ST group (*n* = 49)		*p*
QuickDASH score				
At 3 months	35.38 ± 4.49	32.88 ± 4.38		0.006*
At 12 months	16.22 ± 5.31	14.82 ± 4.31		0.191
PRWE score				
At 3 months	31.81 ± 5.46	28.90 ± 4.92		0.003*
At 12 months	18.26 ± 4.89	17.61 ± 5.67		0.265
Wrist flexion (°)				
At 3 months	63.27 ± 5.89	65.45 ± 6.54		0.070
At 12 months	70.44 ± 7.07	71.12 ± 6.06		0.686
Wrist extension (°)				
At 3 months	58.60 ± 6.12	57.24 ± 6.20		0.228
At 12 months	64.86 ± 5.92	65.78 ± 6.31		0.409
Pronation (°)				
At 3 months	79.77 ± 5.40	81.35 ± 5.82		0.122
At 12 Months	85.36 ± 5.14	85.53 ± 6.16		0.866
Supination (°)				
At 3 months	74.68 ± 5.76	75.57 ± 5.39		0.457
At 12 months	78.12 ± 4.93	80.14 ± 5.37		0.053
Grip strength (kg)				
At 12 months (fractured side)	21.65 ± 5.27	21.78 ± 5.75		0.836
Non‐fractured side	25.09 ± 6.05	24.59 ± 6.48		0.249
Difference (non‐fractured–fractured)	3.43 ± 0.87	2.81 ± 0.83		0.001*

*Note:* QuickDASH and PRWE scores range from 0 to 100, with higher scores indicating worse functional outcomes. Continuous variables were compared using the independent samples *t*‐test (*t* values are reported).

* *p* < 0.05 was considered statistically significant.

### 3.3 Complications

Complications are summarized in Table 4. In the ST group, implant irritation was observed in 4 patients and was statistically significant (*p* = 0.01). In the CT group, malunion occurred in 6 patients, and this difference was also statistically significant (*p* = 0.047). Two patients in the ST group developed superficial skin infections, both of which were successfully managed with oral antibiotics and local wound care, without progression to deep infection. Postoperative complications, including complex regional pain syndrome (CRPS), tendon‐related problems, neuropathy, and superficial infection, were observed in a limited number of patients. However, no statistically significant differences were detected between the conservative treatment and surgical treatment groups regarding complication rates (Table 4).

**TABLE 4:** Distribution of complications by treatment group.

Variable	CT group (*n* = 78)	ST group (*n* = 49)		*p*
CRPS, *n* (%)	0 (0.0%)	2 (4.1%)		0.072
Radioulnar instability, *n* (%)	3 (3.8%)	0 (0.0%)		0.165
Implant irritation, *n* (%)	0 (0.0%)	4 (8.2%)		0.010^a^
Superficial skin infection, *n* (%)	0 (0.0%)	2 (4.1%)		0.072
Tendon‐related problems, *n* (%)	0 (0.0%)	2 (4.1%)		0.072
Neuropathy, *n* (%)	0 (0.0%)	1 (2.0%)		0.205
Malunion, *n* (%)	6 (7.7%)	0 (0.0%)		0.047^a^

^a^ *p* < 0.05 was considered statistically significant. Pearson Chi‐square test (with exact *p* values when appropriate) was used.

### 3.4 Predictors of Surgical Intervention

Multivariable logistic regression analysis was performed to identify factors independently associated with conversion to surgical treatment (Table 5). Fracture severity, as defined by AO classification, was a significant predictor of surgical treatment, with AO/OTA C2 fractures demonstrating a higher likelihood of conversion compared to C1 fractures (OR = 2.50; 95% CI, 1.03–6.09; *p* = 0.043). Reduction quality emerged as the strongest independent predictor of surgical conversion (*p* < 0.001). Compared with anatomic reduction, insufficient reduction at level 1 was associated with a more than fourfold increase in the odds of surgical treatment (OR = 4.17; 95% CI, 1.48–11.72; *p* = 0.007), while insufficient reduction at level 2 demonstrated an even greater effect, with an approximately fourteenfold increase in the likelihood of surgical conversion (OR = 13.97; 95% CI, 3.80–51.37; *p* < 0.001). Among post‐reduction radiographic parameters, increased ulnar variance was independently associated with a higher likelihood of surgical treatment (OR = 1.91; 95% CI, 1.16–3.12; *p* = 0.010). In contrast, volar tilt, radial inclination, and articular step‐off measured after reduction were not significantly associated with surgical conversion. Patient‐related factors, including age, sex, trauma mechanism, and reduction loosening time, were not independently associated with the need for surgical treatment in the multivariable model.

**TABLE 5:** Logistic regression analysis of factors associated with surgical treatment.

Variable	OR	95% CI lower	95% CI upper	*p*
Gender (female vs. male)	0.53	0.20	1.39	0.195
Trauma mechanism (high vs. low energy)	0.56	0.16	2.05	0.385
AO classification (C2 vs. C1)	2.50	1.03	6.09	0.043
Reduction quality (overall)	—	—	—	< 0.001
Not enough vs. anatomic (level 1)	4.17	1.48	11.72	0.007
Not enough vs. anatomic (level 2)	13.97	3.80	51.37	< 0.001
Age (per year increase)	0.99	0.92	1.05	0.634
Reduction loosening time (per week)	0.76	0.40	1.42	0.385
Volar tilt after reduction (°)	1.01	0.87	1.17	0.926
Radial inclination after reduction (°)	0.95	0.83	1.09	0.487
Ulnar variance after reduction	1.91	1.16	3.12	0.010
Articular step‐off after reduction (mm)	0.99	0.40	2.41	0.976

## 4 Discussion

### 4.1 Main Findings of the Study

In this study, short‐ and long‐term outcomes of surgical and conservative treatments were compared in patients aged 65 and older with distal radius fractures and subsequent loss of reduction. At the three‐month follow‐up, the surgical group demonstrated significantly better radiographic alignment than the conservative group in terms of volar tilt, radial inclination, and ulnar variance. During the same period, functional outcomes were also more favorable in the surgical group, as indicated by lower QuickDASH and PRWE scores. In terms of complications, implant irritation was more frequently observed in the surgical group (8.2%), while the conservative group had a significantly higher rate of malunion (7.7%). According to multivariate logistic regression analysis, AO/OTA type 23‐C2 fractures and inadequate reduction quality were identified as independent predictors of surgical intervention. Conversely, increased ulnar variance was independently associated with a higher likelihood of conversion to surgical treatment.

### 4.2 Functional and Radiological Outcomes

When both functional and radiological findings were considered, the surgical group demonstrated significantly better outcomes than the conservative group at the 3‐month follow‐up, including lower QuickDASH and PRWE scores and improved radiological parameters such as volar tilt, radial inclination, and ulnar variance. These early advantages are consistent with previous studies highlighting the role of open reduction and internal fixation in restoring and maintaining joint alignment [3, 11, 20]. However, these differences were no longer observed at the 12‐month follow‐up with respect to functional outcomes. This finding suggests that the early benefits associated with surgical intervention may diminish over time in elderly patients. Possible explanations include age‐related factors such as lower functional demands, reduced neuromuscular adaptability, and the development of compensatory movement strategies. Similar trends have been reported in previous studies, indicating that the initial advantages of surgical treatment tend to converge with conservative outcomes in the long term [4, 11, 12]. Further prospective studies are warranted to better elucidate the mechanisms underlying this temporal pattern.

### 4.3 Complications

When examining the complication profile, the most frequently observed issue in the surgical group was implant irritation, reported in 8.2% of patients. This is a well‐recognized complication associated with volar plating, underscoring the importance of careful patient selection and thorough preoperative counseling regarding potential implant‐related symptoms when considering surgical stabilization. In the conservative group, the most common complication was malunion (7.7%), which was particularly noted in cases with persistent dorsal angulation and could result in lasting deficits in grip strength and wrist range of motion. Such complications may have a direct impact on functional outcomes and should be considered key factors when determining the appropriate treatment strategy. Given that major complication rates following surgical treatment have been reported in the literature to range between 10% and 15%, the rates observed in our study appear consistent with previously published data [10, 21, 22]. On the other hand, the majority of complications in both treatment groups were minor in nature, and no severe events such as CRPS, nerve injury, or infection were encountered. While observational, these findings suggest that appropriate patient selection and treatment performed by experienced teams may help minimize the risk of complications.

### 4.4 Predictors of Surgical Intervention

According to the multivariate logistic regression analysis, the presence of an AO/OTA 23‐C2 fracture and inadequate post‐reduction alignment were identified as independent predictors significantly associated with surgical intervention. Notably, the likelihood of surgery was approximately 15 times higher in cases with poor reduction quality (OR: 15.1; 95% CI: 4.4–51.7), highlighting the strength of this association. This finding indicates that treatment decisions are influenced not only by fracture morphology but also by the radiological quality achieved after reduction. Although thresholds such as volar tilt < 5° have been proposed in the literature as indicators of inadequate reduction, our analysis did not identify volar tilt as an independent determinant of surgical decision‐making [23]. In contrast, increased ulnar variance was independently associated with a higher likelihood of surgical intervention (OR: 1.91; *p* = 0.010), suggesting that progressive positive ulnar variance may reflect mechanical instability following initial reduction. While previous studies have suggested that certain degrees of radiographic deviation may not always correlate with poorer functional outcomes in elderly patients [10, 13, 24], our findings indicate that ulnar variance remains a relevant radiographic factor influencing treatment decisions in this specific clinical context. Therefore, treatment decisions should not rely solely on isolated radiographic thresholds but should integrate overall alignment stability, fracture characteristics, and individualized clinical assessment.

### 4.5 Limitations and Strengths

This study has several limitations. First, due to its retrospective design, treatment allocation was not randomized. As such, the decision to proceed with surgical or conservative management may have been influenced by unpredictable factors, including clinical tendencies, surgeon discretion, and patient preference. This introduces a risk of selection bias, particularly when interpreting the effects attributable to each treatment modality. Second, the grouping of different surgical techniques under a single category may reduce the homogeneity of treatment effects and limit the ability to draw subgroup‐specific clinical conclusions. Additionally, limited sample sizes in some AO/OTA subgroups restricted statistical power, potentially masking clinically relevant differences. In terms of outcome measures, patient‐reported tools such as the QuickDASH and PRWE are subject to response bias and may not fully capture objective functional outcomes. Moreover, evaluations conducted only at 3 and 12 months may not detect interim changes or late‐emerging complications. In addition, detailed radiological parameters were not systematically evaluated at the 12‐month follow‐up. Therefore, conclusions regarding long‐term radiological outcomes should be interpreted with caution. Another limitation of this study is that rehabilitation strategies were not fully standardized or quantitatively analyzed. Although all patients received the same basic home‐based rehabilitation instructions, referral to supervised physiotherapy was made on an individual basis according to clinical need. Due to the retrospective nature of the study, detailed data regarding the frequency, duration, and intensity of supervised rehabilitation were not consistently available and therefore could not be included in the analysis.

Despite these limitations, the present study has several notable strengths. A key strength is its focus on a clinically relevant and well‐defined patient subgroup—elderly individuals who developed loss of reduction after an initially attempted closed reduction—an area that has been insufficiently addressed in the existing literature. The multicenter design and the inclusion of a relatively homogeneous elderly population enhance the external validity of the findings and improve their applicability to real‐world clinical practice. From a clinical perspective, the results provide practical insights into decision‐making in elderly patients with distal radius fractures complicated by loss of reduction. In patients with AO/OTA 23‐C2 fracture patterns or progressive deterioration in post‐reduction alignment during follow‐up, surgical stabilization may be considered due to its short‐term radiological and functional advantages. However, in patients with lower functional demands or increased surgical risk, continued conservative management remains a reasonable option, as comparable outcomes may be achieved in the long term. Accordingly, treatment strategies should be individualized by integrating fracture characteristics, stability during follow‐up, and patient‐specific goals.

## 5 Conclusion

In this multicenter study on elderly patients with distal radius fractures complicated by loss of reduction, surgical treatment demonstrated superior early outcomes in both radiological alignment and functional recovery compared to conservative management. Fracture type AO/OTA 23‐C2 and inadequate post‐reduction alignment emerged as predictive factors for surgical intervention. Notably, poor reduction quality and increased ulnar variance were associated with a higher likelihood of conversion to surgical treatment. However, the early advantage of surgical treatment diminished over time, with both treatment groups showing comparable functional outcomes at 12 months. These findings highlight the critical importance of fracture morphology and reduction quality in guiding treatment decisions, while also emphasizing the need to individualize management based on patient‐specific characteristics and functional expectations.

## Author Contributions

**Bekir Karagoz:** conceptualization, investigation, funding acquisition, writing – original draft, writing – review and editing, visualization, validation, methodology. **Hünkar Cagdas Bayrak:** methodology, visualization, writing – review and editing, validation, resources, supervision, data curation. **Durmus Ekin Dincer:** resources, supervision, data curation, software, formal analysis, project administration.

## Funding

The authors have nothing to report.

## Disclosure

Authorship Declaration: We hereby declare that all authors listed meet the authorship criteria as defined by the latest guidelines of the International Committee of Medical Journal Editors (ICMJE). All authors have made substantial contributions to the conception, design, data collection, analysis, and/or interpretation of the study, have been involved in drafting or critically revising the manuscript, and have approved the final version for submission. All authors agree to be accountable for the accuracy and integrity of the work.

## Ethics Statement

This study was performed in line with the principles of the Declaration of Helsinki. Approval was granted by Eskisehir City Hospital Non‐Interventional Clinical Research Ethics Committee (Approval Date: April 25, 2025; No: ESH/BAEK 2025/149).

## Conflicts of Interest

The authors declare no conflicts of interest.

## Data Availability

The data that support the findings of this study are available from the corresponding author upon reasonable request.
